# Feasibility of utilizing ultra-low-dose contrast medium for pancreatic artery depiction using the combination of advanced virtual monoenergetic imaging and high-concentration contrast medium: an intra-patient study

**DOI:** 10.1186/s13244-021-01079-2

**Published:** 2021-11-12

**Authors:** Juan Li, Yu-hong Wang, Fu-ling Zheng, Xin-yue Chen, Yun Lin, Cai-rong Zhu, Yi-fan Wu, Qiang Xu, Zheng-yu Jin, Hua-dan Xue

**Affiliations:** 1 Department of Radiology, State Key Laboratory of Complex Severe and Rare Disease, Peking Union Medical College Hospital, Chinese Academy of Medical Sciences, Peking Union Medical College, Beijing, China; 2 CT Collaboration, Siemens-Healthineers, China; 3 Global Medical and Regulatory Affairs, Bracco Imaging Medical Technologies Co., Ltd, Shanghai, China; 4 Department of Epidemiology and Health Statistics, West China School of Public Health and West China Fourth Hospital, Sichuan University, Chengdu, China; 5 Department of General Surgery, Peking Union Medical College Hospital, Chinese Academy of Medical Sciences, Peking Union Medical College, Beijing, China

**Keywords:** Computed tomography angiography, Pancreas, Arteries, Contrast media

## Abstract

**Objectives:**

The least amount of contrast medium (CM) should be used under the premise of adequate diagnosis. The purpose of this study is to evaluate the feasibility of utilizing ultra-low-dose (224 mgI/kg) CM for pancreatic artery depiction using the combination of advanced virtual monoenergetic imaging (VMI+) and high-concentration (400 mgI/mL) CM.

**Materials and methods:**

41 patients who underwent both normal dose CM (ND-CM, 320 mgI/kg) and low dose CM (LD-CM, 224 mgI/kg) thoracoabdominal enhanced CT for tumor follow-up were prospectively included. The VMI+ at the energy level of 40-kev for LD-CM images was reconstructed. CT attenuation, signal-to-noise ratios (SNRs), and contrast-to-noise ratios (CNRs) of the abdominal artery, celiac artery, and superior mesenteric artery (SMA) and qualitative scores of pancreatic arteries depiction were recorded and compared among the three groups (ND-CM, LD-CM, and VMI+ LD-CM images). ANOVA and Friedman tests were used for statistical analysis.

**Results:**

All quantitative and qualitative parameters on LD-CM images were lower than that on ND-CM images (all *p* < 0.01). There were no significant differences of all arteries’ qualitative scores between ND-CM and VMI+ LD-CM images (all *p* > 0.05). VMI+ LD-CM images had the highest mean CT and CNR values of all arteries (all *p* < 0.0001). The CM volume was 52.6 ± 9.4 mL for the ND-CM group and 37.0 ± 6.7 mL for the LD-CM group.

**Conclusion:**

Ultra-low-dose CM (224 mgI/kg) was feasible for depicting pancreatic arteries. Inferior angiographic image quality could be successfully compensated by VMI+ and high-concentration CM.

## Key Points

- Ultra-low dose (224 mgI/kg) contrast medium could be used for pancreatic artery depiction.
- Reduced image quality could be compensated by VMI+ technique.
- This imaging strategy can save half of contrast medium costs.

## Background

Abdominal CTA is widely used to assess vascular disorders [1] and guide surgical planning for patients with tumors [2]. However, the contrast medium (CM) dosage is associated with contrast-induced nephropathy and other chemotoxic reactions [3, 4]. The European Society of Urogenital Radiology guidelines recommend that the least amount of CM should be used under the premise of adequate diagnosis [5]. Nevertheless, sufficient CM is necessary for the depiction of small vessels [6]. Pancreas is a hypervascular organ, and the average diameter of the lumen of pancreatic arteries of approximately 2 mm [7]. Although the resectability of pancreatic carcinoma depends on the main vessels around the pancreas, pancreatic arteries have been reported to correlate with blood loss in surgeries, which is a known risk factor for postoperative complications [8]. For instance, for pancreaticoduodenectomy using the artery as the first approach, the preoperative identification of inferior pancreaticoduodenal artery (IPDA) on CT angiography allows IDPA ligation in a shorter time. Moreover, the successful ligation of IDPA before an efferent vein resolves the main cause of intraoperative blood loss in pancreaticoduodenectomy. In addition, for more dedicated surgeries such as pancreas transplants, understanding the different pancreatic artery types is important to prevent possible complications [9]. Therefore, using techniques for good pancreatic arteries visualization that allows the least amount of CM is of great importance.

Methods for improving abdominal CTA image quality acquired using a reduced dose of CM include virtual monochromatic imaging (VMI) [6], test-bolus injection [10], low-tube-voltage imaging [11, 12], CTA individual-delay bolus tracking [13], multiphasic injection [14] and high-concentration CM imaging [15]. Notably, the abovementioned methods are widely used for CM dose reduction in the imaging of the abdominal aorta and its main branches. Only a few studies have focused on small arteries. According to Sugawara H et al. [6], though half-iodine dose (300 mgI/kg) VMI could depict large vessels comparable to full-iodine dose (600 mgI/kg) conventional CT, VMI failed to visualize small arteries clearly. The concentration of CM in their study was 300 mgI/mL. On the contrary, in another study, Holalkere NS et al. [15] used high-concentration CM (370 mgI/mL) and demonstrated that the third to the fifth order branches of the superior mesenteric artery (SMA) could be depicted clearly with 20% less iodine dose (full-iodine dose: 600 mgI/kg, 20% less-iodine dose: 480 mgI/kg).

As for a fixed volume of CM and a constant injection rate, a higher concentration of CM could produce a greater magnitude of aortic enhancement by increasing the iodine delivery rate (IDR). Moreover, with the advanced virtual monoenergetic imaging (VMI+) technique, images reconstructed at the energy level of 40 keV, which is the closest possible to the K-edge of iodine (33 keV), have better quality than images reconstructed using the VMI technique [16]. In this study, we hypothesized that by combining high-concentration CM (400 mgI/mL) and VMI+, ultra-low-dose CM (224 mgI/kg) abdominal CTA images could depict both large vessels and pancreatic arteries clearly.

The purpose of this study was to evaluate the feasibility of ultra-low-dose CM (224 mgI/kg) for depicting abdominal arteries, especially for pancreatic artery visualization, using the combination of VMI+ and high-concentration CM (400 mgI/mL).

## Materials and methods

This prospective study was approved by the Institutional Review Board of Peking Union Medical College Hospital. Written informed consent was obtained from all patients.

### Subjects

From April 2019 to May 2020, 124 consecutive patients who received follow-up thoracoabdominal enhanced CT scans at our hospital for post-treatment tumor surveillance were enrolled in this study. The exclusion criteria were as follows: (1) patients with contraindications to intravenous administration of iodine CM; (2) patients with pancreatic mass, with consideration that it might affect pancreatic arteries depiction; (3) structural changes of abdominal blood vessels caused by surgery; (4) patients whose follow-up CTs were lost in our hospital. Finally, 41 patients were included in this study after excluding 3 patients with structural changes in the abdominal blood vessels caused by surgery (2 patients underwent total gastrectomy and 1 patient underwent splenectomy), 2 patients with pancreatic mass, and 78 patients with lost follow-up CT.

### Study design

This was an intra-patient study in which each patient consecutively received both normal-dose CM (ND-CM, 400 mgI/mL, 0.8 mL/kg, and 320 mgI/kg) and low-dose CM (LD-CM, 400 mgI/mL, 0.56 mL/kg, and 224 mgI/kg) in a random order for the first scan and follow-up to minimize patient factors that would affect artery visualization. High-concentration CM was used in our study to achieve high IDR under a constant injection rate. The CM concentration and injection rate, which determine the IDR, were the same in the two groups such that the CM dose would be the only factor affecting the magnitude of artery enhancement. The rationale for the low-dose group design was that we assumed that a 30% reduction of the normal-dose group was feasible, as previous low-dose studies of low kVp [17] or dual-energy [18, 19] enabled a 50% reduction of iodine load at most.

### CT examination

All patients underwent thoracoabdominal enhanced CT using a 192-detector MDCT scanner (Siemens Somatom Force; Siemens, Forchheim, Germany). The CM was warmed to achieve normal body temperature (37 °C) before injection. After performing topogram and nonenhanced scan, patients received the CM at a rate of 4 mL/s, followed by 40 mL of saline at the same rate. Using bolus tracking, the arterial phase was initiated for 5 s for the LD-CM group and 6 s for the ND-CM group after achieving the threshold density of 100 HU in the aortic arch. The scanning parameters were as follows: 100 kV/250 (ref. mAs for tube A); 150 kV/125 (ref. mAs for tube B); pitch, 1.2; collimation, 128 × 0.6 mm; rotation time, 0.5 s; kernel Br40f, 0.6 mm slice thickness and 0.4 mm slice increment were used for vascular evaluation.

### Image post-processing

Arterial phase images were transferred to a workstation (syngo.via, version VB10, Siemens Healthineers) for analysis. VMI+ images at the energy level of 40 keV of the LD-CM scanning were reconstructed. The coronal maximum intensity projection (MIP) images (10-mm thickness, 1-mm interval) of the ND-CM scanning, LD-CM scanning, and VMI+ images of the LD-CM scanning were reconstructed for analysis.

### Image assessment

#### Quantitative analysis of abdominal aorta and its main branches

**Fig. 1:**
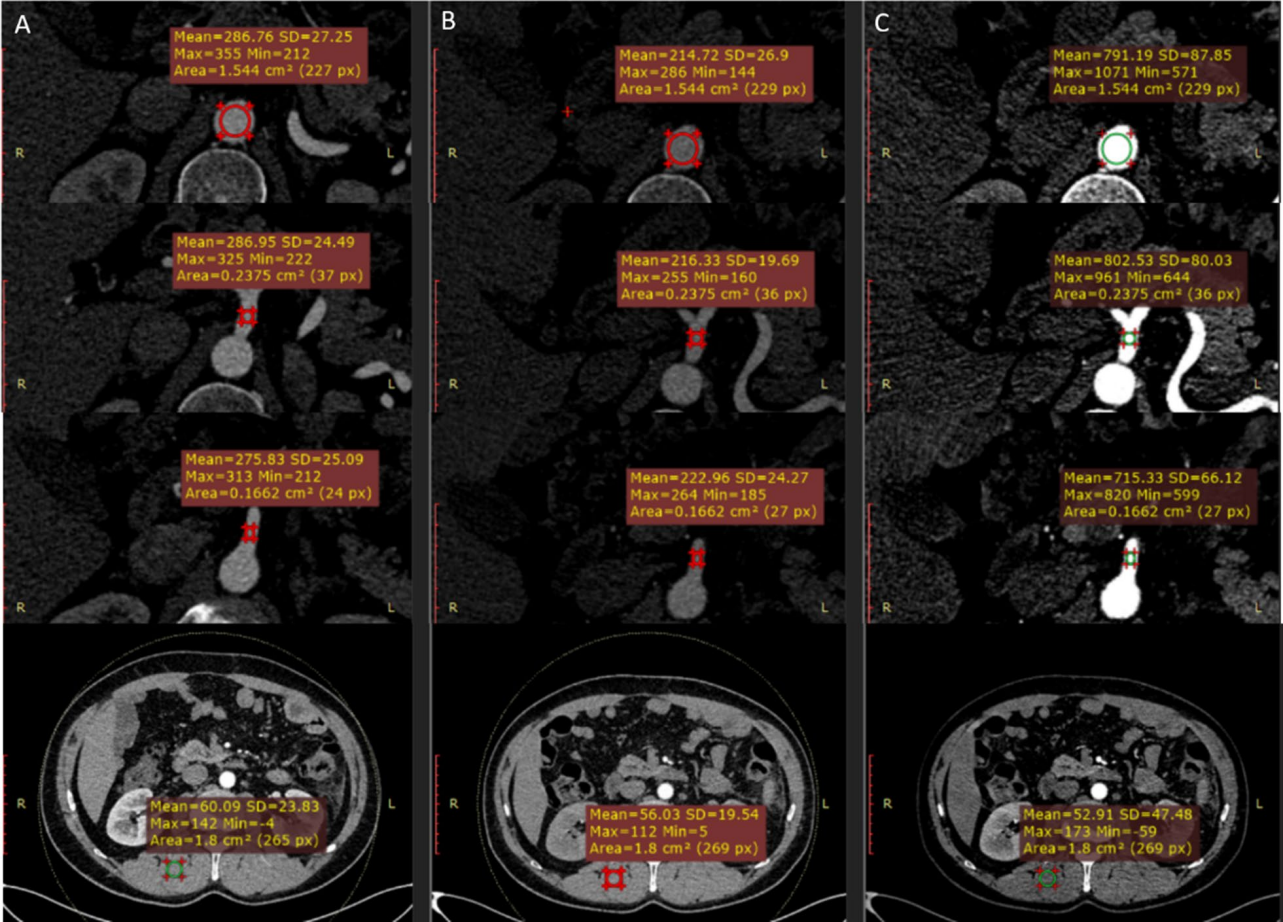
Quantitative analysis of main branches of abdominal arteries. **a**–**c** ND-CM (0.8 mL/kg, 400 mgI/mL, and 320 mgI/kg), LD-CM (0.56 mL/kg, 400 mgI/mL, and 224 mgI/kg), and VMI+ LD-CM images in the same patient. Images show the ROI for attenuation measurement at the level of the aorta (first line), celiac artery (second line), superior mesenteric artery (SMA, third line), and right erector spinae muscle (last line). *ND-CM* normal-dose contrast medium, *LD-CM* low-dose contrast medium, *VMI*+ advanced virtual monoenergetic imaging

Circular regions of interest (ROIs) were drawn by one radiologist on the abdominal aorta at the level between the celiac artery orifice and the SMA, the celiac artery, SMA and right erector spinae muscle on axial images (Fig. 1) of the ND-CM scanning, LD-CM scanning, and VMI+ images of the LD-CM scanning using an independent DICOM viewer (Medixant. RadiAnt DICOM Viewer. Version 4.6.9. Oct 25, 2018. URL: https://www.radiantviewer.com). The ROIs were carefully drawn to avoid the wall, the calcification or noncalcified plaque of the vessels, and artifacts. The size and shape of the ROIs were kept constant using the copy-and-paste function. The average and standard deviation (SD) values of CT attenuation of all ROIs were recorded. The SD of the right erector spinae muscle was used to define the image noise. Signal-to-noise ratios (SNRs) of vessels were calculated by Mean_vessel_(HU)/SD_vessel_(HU). Contrast-to-noise ratios (CNRs) of vessels were calculated as follows: (Mean_vessel_(HU) − Mean_muscle_(HU))/SD_muscle_(HU).

#### Qualitative analysis of abdominal aorta, its main branches, and pancreatic arteries

The axial and coronal multiplanar reformation images (thickness: 0.6 mm) and coronal MIP images of ND-CM scanning, LD-CM scanning, and VMI+ images of LD-CM scanning were read by two radiologists (one with 4 years of experience and the other with more than 10 years of experience in abdominal imaging) using the same DICOM viewer. The readers were blinded to the clinical information or identification of the reconstruction method. The three groups of images from each patient were randomized, and the readers evaluated one set at a time in a random order, with a time interval of 2 weeks. The visualization of the abdominal artery, celiac artery, and SMA were graded using a 4-point scale: 1 = no depiction, 2 = faint depiction, 3 = good depiction, and 4 = excellent depiction. Pancreatic arteries (posterior superior pancreaticoduodenal artery [PSPDA]; anterior superior pancreaticoduodenal artery [ASPDA]; inferior pancreaticoduodenal artery [IPDA]; anterior inferior pancreaticoduodenal artery [AIPDA]; posterior inferior pancreaticoduodenal artery [PIPDA]; dorsal pancreatic artery [DPA]; transverse pancreatic artery [TPA]; great pancreatic artery [GPA]; and caudal pancreatic artery [CPA]) were graded using a 4-point scale: 0 = not visible, 1 = partially visible, 2 = partially invisible or slightly irregular appearance, and 3 = completely visible [6]. First, a qualitative scoring of pancreatic arteries was performed on the coronal MIP images. If considered invisible, the axial and coronal images were then used to confirm that the pancreatic arteries were invisible. Any inconsistent results were discussed by the two radiologists to make a final decision. The luminal diameters of pancreatic arteries were then measured using multiplanar reconstruction following curvilinear planes [7, 20]. The depiction rate of pancreatic arteries was calculated by dividing the number of patients with visible pancreatic arteries by the total number of patients.

### Statistical analysis

All statistical analyses were performed using SAS software version 9.4 (SAS Institute, Inc., Cary, NC, USA). Continuous variables with normal distribution were expressed as mean ± SD. Categorical and continuous variables without normal distribution were displayed in median (quartile 2, quartile 3). The paired t-test or Wilcoxon test was used to compare the differences in patient demographics and contrast dose parameters between ND-CM and LD-CM groups. When comparing the differences in parameters among the three groups, if continuous variables satisfied both normal distribution and homogeneity of variance, a randomized block ANOVA was used to compare intergroup differences; otherwise, the Friedman test was used. The differences of qualitative scores among the three groups were compared using the Friedman test. A *p* value < 0.05 were considered statistically significant. Interobserver agreement for qualitative evaluation was assessed by Cohen’s kappa (*κ*) analysis: *κ* of < 0 indicative poor agreement, 0–0.20 slight agreement, 0.21–0.40 fair agreement, 0.41–0.60 moderate agreement, 0.61–0.80 substantial agreement, and 0.81–1 almost perfect agreement.

## Results

### Patient population

A total of 41 patients including 26 males and 15 females (mean age, 56 ± 13 years; age range, 24–77 years) were included in this study. Primary diseases among the patients were colorectal cancer (*n* = 14), lung cancer (*n* = 10), esophageal cancer (*n* = 3), gastric cancer (*n* = 2), thymic carcinoma (*n* = 2), breast cancer (*n* = 1), peritoneal mesothelioma (*n* = 1), testicular cancer (*n* = 1), stromal sarcoma (*n* = 1), leiomyosarcoma (*n* = 1), hypopharyngeal carcinoma (*n* = 1), angioendothelioma (*n* = 1), rectal stromal tumor (*n* = 1), appendix mucinous adenocarcinoma (*n* = 1), and soft tissue sarcoma (*n* = 1). The interval between the first examination and the follow-up was 135 ± 78 days. The data on patient demographics and contrast dose are listed in Table 1.

**Table 1:** Patient demographics (*n* = 41) and contrast dose

Variables	ND-CM	LD-CM	*p* value
Age (y)	56.1 ± 13.1	56.0 ± 13.0	0.1250
Sex			
Male	26	26	–
Female	15	15	–
Weight (kg)	65.74 ± 11.81	66.00 ± 11.99	0.7614
Height (cm)	165.59 ± 8.74	165.59 ± 8.58	1.0000
Body mass index (kg/cm^2^)	24.07 ± 3.59	24.08 ± 3.63	0.3009
Iodine dose (mgI/kg)	320	224	–
Iodine volume	52.59 ± 9.44	36.96 ± 6.71	< 0.0001
Capacity per bottle (mL)	50	50	–

Data are means ± standard deviations

The difference between ND-CM group and LD-CM group by using the paired t test or Wilcoxon signed test. *p* < 0.05 is considered statistically significant

ND-CM: normal dose contrast medium (320 mgI/kg), LD-CM: low dose contrast medium (224 mgI/kg)

### Quantitative analysis of abdominal aorta and its main branches

Mean CT and CNR values of all vessels were the highest in VMI+ LD-CM images, followed by ND-CM images, and the lowest in LD-CM images (all *p* < 0.0001). The SNR values of all vessels on VMI+ LD-CM and ND-CM images were higher than those in LD-CM images (all *p* < 0.05). The SNR of the aorta in VMI+ LD-CM images were lower than those in ND-CM images (*p* = 0.0465), whereas there was no significant difference in the SNRs of the celiac artery and SMA between ND-CM and VMI+ LD-CM images (*p* = 0.6911 and 1.0000, respectively). The image noise in VMI+ LD-CM images was significantly higher than that of ND-CM and LD-CM images (both *p* < 0.0001) (Table 2).

**Table 2:** Results of quantitative analysis

Parameters	ND-CM images*	LD-CM images*	LD-CM VMI+ images*	*p*_1_ value^†^	*p*_2_ value^†^	*p*_3_ value^†^
*Average value (HU)*						
Aorta	337.8 ± 55.36	260.16 ± 73.08	1029.76 (735.08 ,1223.75)	< 0.0001	< 0.0001	< 0.0001
Celiac artery	343.42 ± 56.01	285.32 (213.88, 325.31)	1078.28 (817.35, 1219.92)	< 0.0001	< 0.0001	< 0.0001
SMA	346.65 ± 60.24	285.27 (211.30, 336.15)	1034.77 (713.07, 1227.04)	< 0.0001	< 0.0001	< 0.0001
Right Erector spinae muscles	53.76 ± 6.26	53.68 ± 4.98	66.79 ± 10.60	0.3471	< 0.0001	< 0.0001
*Image noise (HU)*						
Right Erector spinae muscles	19.99 ± 3.67	19.79 ± 3.33	44.15 ± 9.41	0.5136	< 0.0001	< 0.0001
*Signal to noise ratio (SNR)*						
Aorta	13.38 ± 2.92	10.38 ± 2.64	12.06 ± 3.29	< 0.0001**	0.0465**	0.0113**
Celiac artery	15.27 ± 4.10	11.94 ± 3.64	13.67 (10.27, 15.27)	< 0.0001	0.6911	< 0.0001
SMA	15.1 ± 3.45	11.58 ± 3.55	14.25 ± 5.09	< 0.0001	1.0000	< 0.0001
*Contrast to noise ratio (CNR)*						
Aorta	14.57 ± 3.64	10.46 ± 3.81	20.71 ± 7.30	< 0.0001	< 0.0001	< 0.0001
Celiac artery	14.86 ± 3.71	10.85 ± 4.00	21.23 ± 7.17	< 0.0001	< 0.0001	< 0.0001
SMA	15.03 ± 3.94	10.86 ± 4.06	21.00 ± 7.30	< 0.0001	< 0.0001	< 0.0001

Data are means ± standard deviations or Median (P25, P75)

*ND-CM: normal dose contrast medium (320 mgI/kg), LD-CM: low dose contrast medium (224 mgI/kg), VMI+ LD-CM images: advanced virtual monoenergetic imaging of LD-CM images

**p value was calculated by *R*

^†^*P*_1_: the difference between ND-CM images and LD-CM images; *P*_2_: the difference between ND-CM images and VMI+ LD-CM images; *P*_3_: the difference between LD-CM images and VMI+ LD-CM images by using the randomized block ANOVA analysis or Friedman test. *p* < 0.05 is considered statistically significant

### Qualitative analysis of abdominal aorta, its main branches and pancreatic arteries

There was substantial to perfect agreement between the two readers in qualitative analysis of the depiction of all arteries (mean *κ* = 0.8465, range, 0.6997–1.0000).

### Depiction rate and lumen diameter of pancreatic arteries

The depiction rates of all pancreatic arteries were higher in ND-CM images than in LD-CM and VMI+ LD-CM images (Table 3). Moreover, ND-CM images could depict all arteries visualized on LD-CM and VMI+ LD-CM images.

**Table 3:** Depiction rates and lumen diameters of pancreatic arteries

Artery	lumen diameter(mm)	Depiction rates		
		ND-CM images^§^	LD-CM images^§^	LD-CM VMI+ images^§^
PSPDA*	1.6 (1.5, 1.8)	100.00% (41/41)	95.12% (39/41)	97.56% (40/41)
ASPDA*	1.7 ± 0.3	100.00% (41/41)	90.24% (37/41)	92.68% (38/41)
IPDA*	1.9 ± 0.4	60.98% (25/41)	51.22% (21/41)	51.22% (21/41)
AIPDA*	1.4 ± 0.3	80.49% (33/41)	73.17% (30/41)	73.17% (30/41)
PIPDA*	1.3 ± 0.3	87.80% (36/41)	80.49% (33/41)	80.49% (33/41)
DPA*	1.7 ± 0.4	87.80% (36/41)	78.05% (32/41)	78.05% (32/41)
TPA*	1.5 ± 0.3	82.93% (34/41)	70.73% (29/41)	70.73% (29/41)
GPA*	1.5 (1.3, 1.8)	97.56% (40/41)	85.37% (35/41)	85.37% (35/41)
CPA*	1.3 (1.2, 1.4)	65.85% (27/41)	56.10% (23/41)	56.10% (23/41)

*PSPDA posterior superior pancreaticoduodenal artery, *ASPDA* anterior superior pancreaticoduodenal artery, *IPDA* inferior pancreaticoduodenal artery, *AIPDA* anterior inferior pancreaticoduodenal artery, *PIPDA* posterior inferior pancreaticoduodenal artery, *DPA* dorsal pancreatic artery, *TPA* transverse pancreatic artery, *GPA* great pancreatic artery, *CPA* caudal pancreatic artery

^§^*ND-CM* normal dose contrast medium (320 mgI/kg), *LD-CM* low dose contrast medium (224 mgI/kg), *VMI*+ *LD-CM images* advanced virtual monoenergetic imaging of LD-CM images

The depiction rate of pancreatic arteries was calculated by dividing the number of patients with visible pancreatic arteries by the total number of patients

### Qualitative scores of the abdominal aorta, its main branches and pancreatic arteries

**Fig. 2:**
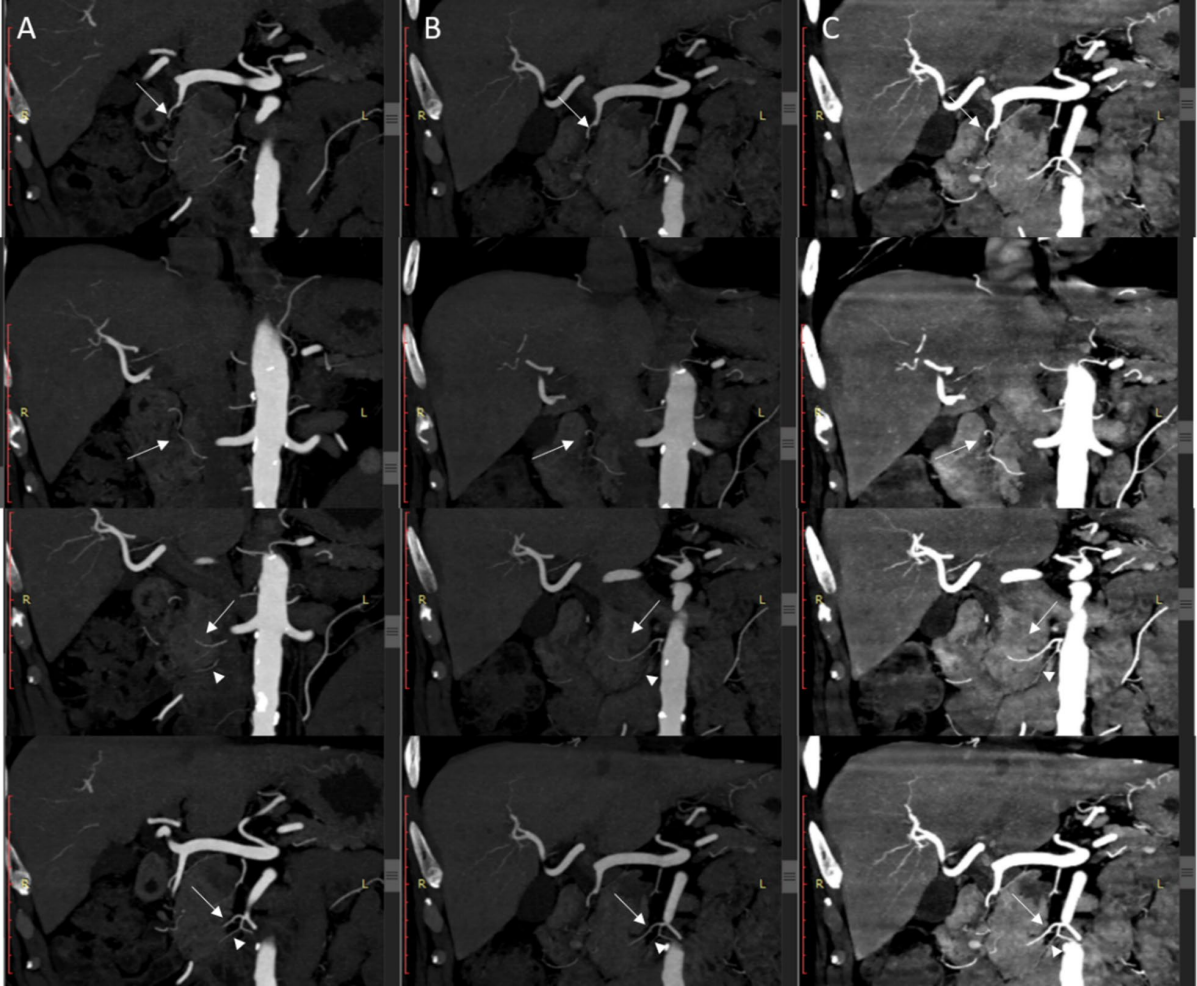
Depiction of pancreatic arteries in ND-CM, LD-CM, and VMI+ LD-CM images. **a**–**c** The posterior pancreaticoduodenal artery arch of one patient who underwent ND-CM (0.8 mL/kg, 400 mgI/mL, and 320 mgI/kg), LD-CM (0.56 mL/kg, 400 mgI/mL, and 224 mgI/kg), and VMI+ LD-CM using the same window level (300 HU) and width (600 HU). The CT value of the aorta was highest on VMI+ LD-CM images (**c**), lower on ND-CM images (**a**), and lowest on LD-CM images (**b**). The posterior pancreaticoduodenal artery arch (white arrow) can be depicted clearly on all images. The anterior inferior pancreaticoduodenal artery (AIPDA) (arrowhead) on LD-CM images (**b**) cannot be seen clearly, which showed a faint display on VMI+ LD-CM images (**c**) and can be seen clearly on ND-CM images (**a**). *ND-CM* normal-dose contrast medium, *LD-CM* low-dose contrast medium, *VMI*+ advanced virtual monoenergetic imaging

**Fig. 3:**
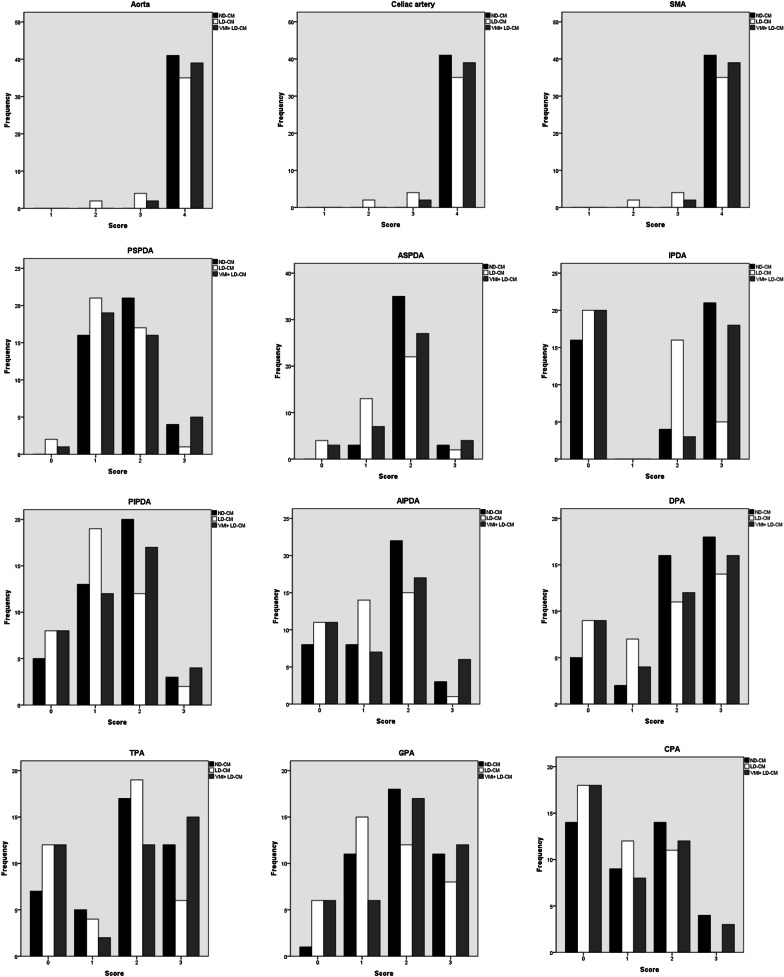
Histograms of the subjective scores of artery depiction in ND-CM, LD-CM, and VMI+ LD-CM images. Histograms show the subjective scores of the abdominal aorta, its main branches, and pancreatic arteries in ND-CM (0.8 mL/kg, 400 mgI/mL, and 320 mgI/kg), LD-CM (0.56 mL/kg, 400 mgI/mL, and 224 mgI/kg), and VMI+ LD-CM images. Visualization of the abdominal artery, celiac artery, and superior mesenteric artery (SMA) were graded using a 4-point scale: 1 = no depiction, 2 = faint depiction, 3 = good depiction, and 4 = excellent depiction. The depiction of pancreatic arteries was graded using a 4-point scale: 0 = not visible, 1 = partially visible, 2 = partially invisible or slightly irregular appearance, and 3 = completely visible. *ND-CM* normal-dose contrast medium, *LD-CM* low-dose contrast medium, *VMI*+ advanced virtual monoenergetic imaging

Qualitative scores of all arteries on VMI+ LD-CM and ND-CM images were higher than those on LD-CM images (all *p* < 0.05), except for the qualitative scores of IPDA and DPA between the VMI+ LD-CM and LD-CM images (*p* = 0.2059 and 0.1038 respectively). There were no significant differences in all arteries between the ND-CM and VMI+ LD-CM images (all *p* > 0.05) (Table 4; Figs. 2, 3).

**Table 4:** Results of qualitative analysis of abdominal aorta, its main branches and pancreatic arteries

Artery	ND-CM^§^	LD-CM^§^	VMI+ LD-CM^§^	*p*_1_ value^†^	*p*_2_ value^†^	*p*_3_ value^†^
Aorta	4 (4,4)	4 (4,4)	4 (4,4)	0.0012	0.5033	0.0087
Celiac artery	4 (4,4)	4 (4,4)	4 (4,4)	0.0012	0.5033	0.0087
SMA	4 (4,4)	4 (4,4)	4 (4,4)	0.0012	0.5033	0.0087
PSPDA*	2 (1,2)	1 (1,2)	2 (1,2)	0.0040	0.5982	0.0172
ASPDA*	2 (2,2)	2 (1,2)	2 (1.5,2)	< 0.0001	0.0799	0.0033
IPDA*	3 (0,3)	2 (0,3)	2 (0,3)	0.0039	0.0929	0.2059
AIPDA*	2 (1,2)	1 (0,2)	2 (0,2)	0.0003	0.7509	0.0008
PIPDA*	2 (1,2)	1 (1,2)	2 (1,2)	0.0005	0.3691	0.0082
DPA*	2 (2,3)	2 (1,3)	2 (1,3)	0.0040	0.1918	0.1038
TPA*	2 (1,3)	2 (0,2)	2 (0,3)	0.0016	0.9163	0.0012
GPA*	2 (1,3)	1 (1,2)	2 (1,3)	0.0008	0.9275	0.0011
CPA*	1 (0,2)	1 (0,2)	1 (0,2)	0.0002	0.1016	0.0230

Data are means ± standard deviations or Median (P25, P75)

*PSPDA posterior superior pancreaticoduodenal artery, *ASPDA* anterior superior pancreaticoduodenal artery, *IPDA* inferior pancreaticoduodenal artery, *AIPDA* anterior inferior pancreaticoduodenal artery, *PIPDA* posterior inferior pancreaticoduodenal artery, *DPA* dorsal pancreatic artery, *TPA* transverse pancreatic artery, *GPA* great pancreatic artery, *CPA* caudal pancreatic artery

^§^*ND-CM* normal dose contrast medium (320 mgI/kg), *LD-CM* low dose contrast medium (224 mgI/kg), *VMI*+ *LD-CM images* advanced virtual monoenergetic imaging of LD-CM images

^†^*P*_1_: the difference between ND-CM images and LD-CM images; *P*_2_: the difference between ND-CM images and VMI+ LD-CM images; *P*_3_: the difference between LD-CM images and VMI+ LD-CM images by using the Friedman test. *p* < 0.05 is considered statistically significant

The visualization of the abdominal artery, celiac artery, SMA were graded using a 4-point scale: 1 = no depiction, 2 = faint depiction, 3 = good depiction and 4 = excellent depiction. The depiction of pancreatic arteries was graded using a 4-point scale: 0 = not visible, 1 = partially visible, 2 = partially invisible or slightly irregular appearance, 3 = completely visible

## Discussion

Our study demonstrated that on the condition of constant IDR, reduced iodine dose could affect the depiction of both large-vessels and pancreatic arteries. However, the VMI+ could reduce the impact. By using the combination of VMI+ and high-concentration CM (400 mgI/mL), ultra-low-dose (224 mgI/kg) CM could be used for the depiction of both large vessels and pancreatic arteries in abdominal CTA without losing the image quality compared to the normal dose (320 mgI/kg). The amount of contrast agent in the ND-CM group is 52.6 ± 9.4 mL, which exceeds the volume of a CM bottle of 50 mL, whereas the volume used in the LD-CM group was less than 50 mL (37.0 ± 6.7 mL). This imaging strategy may reduce the CM dosage and save half of CM costs, especially for patients requiring follow-up by CTA.

The dose of CM in our ND-CM group (320 mgI/kg) was lower than that in the previously reported ND-CM group (600 mgI/kg) [6, 15]. Despite this, the depiction rate of pancreatic arteries in this study was comparable to that reported previously [4, 7]. Notably, the depiction rate for IPDA (60.98%) corroborated with the findings of another study [21], in which 65% of the population had inferior origins of both the anterior and posterior arcades, leaving the SMA as a short common trunk. The difference in the depiction rates of GPA and CPA between Macchi V’s study [7] and our study (73.1% and 96.2% vs. 97.56% and 65.85%, respectively) may be attributed to the different definitions of these arteries. In our study, if multiple arteries arose from the mid-to-distal one-third portion of the splenic artery, the largest one was considered the GPA, but if only one artery was depicted, the location of the artery was used to determine whether it was the GPA or CPA (the artery near the splenic hilum was considered the CPA, and that near the pancreatic body was considered the GPA). To our knowledge, the CM dose used in our study is the lowest dose used for small abdominal arteries; therefore, we called the CM dose of the LD-CM group ultra-low-dose.

The ND-CM (320 mgI/kg) images are quantitatively and qualitatively better than the LD-CM (224 mgI/kg) images, likely due to the following two factors: first, the reduced CM volume leads to reduced enhancement magnitude; second, the reduced injection time results in a narrow peak of vascular enhancement [22], which may be missed in some patients. In our study, although the depiction of pancreatic arteries in four patients was good in the ND-CM images, it was almost invisible in the LD-CM or VMI+ LD-CM images because the enhancement degree of the portal venous system was close to or exceeded that of the proximal artery. The qualitative scores of all arteries in the VMI+ LD-CM group were comparable to those of the ND-CM group (all *p* > 0.05), whereas the depiction rates of some pancreatic arteries in the VMI+ LD-CM images were lower than those in ND-CM images, which implies that VMI+ could improve the visualization of arteries only when they can be depicted on LD-CM images. In addition, this also suggests that the strategy of bolus tracking to capture the accurate time-to-peak of the aorta might not be precise for all patients. Patient-specific individualized trigger-delay technique [23] may be needed to reduce CM in abdominal CTA [13].

The VMI+ technique has been used in CTA of the head and neck [24], aorta [25], thorax, abdomen [26], and lower extremities [27, 28]. Most of these studies focused on improving the image quality. To the best of our knowledge, this technique has not been used as a compensation strategy for the possible inferior image quality of small arteries, such as pancreatic arteries, in CT angiography resulting from low iodine dose. Our results demonstrate that using VMI+ and high-concentration CM (400 mgI/kg), the CM dose could be reduced up to 70% of the ND-CM in the depiction of pancreatic arteries; this finding is in accordance with the findings of a previous study conducted by Holalkere NS et al. [15], which showed that using high-concentration CM (370 mgI/mL), the third to fifth order branches of SMA could be depicted clearly with a 20% less iodine dose. On the other hand, Sugawara H et al. found that VMI failed to provide clear visualization of small arteries by comparing full-iodine-dose (600 mgI/kg) conventional CT and half-iodine-dose (300 mgI/kg) VMI for the depiction of abdominal arteries. Although the depiction of large vessel was comparable between the full-iodine-dose conventional CT and half-iodine-dose VMI [6]. The reasons for this contradiction may be the following: (1) the CM dose reduction in their study is more than that in our study (50% vs. 30%); (2) the CM concentration in their study was less than that in our study (300 mgI/kg vs. 400 mgI/kg); (3) the injection protocol in their study was constant injection duration, whereas that in our study was constant injection rate; thus, reasons 2 and 3 caused different IDRs between their and our studies; (4) the different techniques and energy levels chosen for post-processing (VMI of 52 keV in their study vs. VMI+ of 40 keV in our study). Further, Albrecht MH et al.’s study [26] reported that image contrast and visualization of small artery branches in VMI+ images at the energy level of 40–50 keV are superior to traditional VMI images.

Our study has some limitations: first, some patients with progressive disease or partial response disease were not excluded, and there may be a difference in the artery conditions between the first examination and the follow-up due to different disease conditions. However, we tried to minimize the effect by randomizing the examination sequence and found that the artery depiction was comparable between the first examination and follow-up in these patients; second, the same injection rate rather than the same injection duration was used for ND-CM and LD-CM groups, making it difficult to capture the time-to-peak of the aorta. Therefore, an injection protocol using the same injection duration or scanning protocol for individual-delay bolus tracking may be applied in a future study. Finally, we did not further group the patients according to body mass index (BMI) to assess whether the protocol could be used in patients with high BMI, which may be addressed in the future.

In conclusion, using the combination of VMI+ and high-concentration CM (400 mgI/mL), ultra-low-dose CM (224 mgI/kg) could be used for the depiction of both large vessels and pancreatic arteries in abdominal CTA.

## Data Availability

The datasets used and/or analyzed during the current study are available from the corresponding authors on reasonable request.

## Abbreviations

AIPDA: Anterior inferior pancreaticoduodenal artery
ASPDA: Anterior superior pancreaticoduodenal artery
BMI: Body mass index
CM: Contrast medium
CNR: Contrast-to-noise ratio
CPA: Caudal pancreatic artery
DPA: Dorsal pancreatic artery
GPA: Great pancreatic artery
IDR: Iodine delivery rate
IPDA: Inferior pancreaticoduodenal artery
LD-CM: Low dose CM
MIP: Maximum intensity projection
ND-CM: Normal dose CM
PIPDA: Posterior inferior pancreaticoduodenal artery
PSPDA: Posterior superior pancreaticoduodenal artery
ROI: Regions of interest
SD: Standard deviation
SMA: Superior mesenteric artery
SNR: Signal-to-noise ratio
TPA: Transverse pancreatic artery
VMI: Virtual monochromatic imaging
VMI+: Advanced virtual monoenergetic imaging
